# Dry-Season Snow Cover Losses in the Andes (18°–40°S) driven by Changes in Large-Scale Climate Modes

**DOI:** 10.1038/s41598-019-53486-7

**Published:** 2019-11-18

**Authors:** Raul R. Cordero, Valentina Asencio, Sarah Feron, Alessandro Damiani, Pedro J. Llanillo, Edgardo Sepulveda, Jose Jorquera, Jorge Carrasco, Gino Casassa

**Affiliations:** 1 Universidad de Santiago de Chile, Av. Bernardo O’Higgins 3363, Santiago, Chile; 2 School of Earth, Energy and Environmental Sciences, Stanford University, Stanford, California USA; 3 Center for Environmental Remote Sensing, Chiba University, Chiba, Japan; 4 Centro de Investigación GAIA Antártica, Universidad de Magallanes, Punta Arenas, Chile; 5 Unidad de Glaciología y Nieves, Dirección General de Aguas, Ministerio de Obras Públicas, Santiago, Chile

**Keywords:** Atmospheric science, Cryospheric science

## Abstract

The Andean snowpack is the primary source of water for many communities in South America. We have used Landsat imagery over the period 1986–2018 in order to assess the changes in the snow cover extent across a north-south transect of approximately 2,500 km (18°–40°S). Despite the significant interannual variability, here we show that the dry-season snow cover extent declined across the entire study area at an average rate of about −12% per decade. We also show that this decreasing trend is mainly driven by changes in the El Niño Southern Oscillation (ENSO), especially at latitudes lower than 34°S. At higher latitudes (34°–40°S), where the El Niño signal is weaker, snow cover losses appear to be also influenced by the poleward migration of the westerly winds associated with the positive trend in the Southern Annular Mode (SAM).

## Introduction

Satellite-derived records dating back to the early 1970s show pronounced snow cover (SC) extent reductions in the Northern Hemisphere (NH) especially since 2005^1,2^. Surface-based observations show widespread decreases in snow depth over longer periods of time in North America^3^, Europe^4^ and Asia^5^. These negative trends are expected to continue in the future affecting in turn snowmelt rates and freshwater supply^6,7^.

Excluding Antarctica, most of the snow cover (SC) in the Southern Hemisphere (SH) is restricted to high altitude areas in the Andean region^8,9^. The Andes span more than 7,000 km along western South America and its snowpack is the primary source of water for many communities. Streams deliver the melt water to populated areas of central-western Argentina and central Chile (33°–37°S), where it is important for urban water supply, power generation, and agriculture^10–14^.

Satellite-derived records show that the Andean cryosphere is rapidly decreasing^15–19^. Landsat imagery dating back to 1986 has confirmed a decline in the SC extent in the extratropical Andes^20,21^, For example, the annual minimum SC extent declined about 15% over the period 1986–2011 around latitude 33°S^21^. Satellite estimates retrieved from the Moderate Resolution Imaging Spectroradiometer (MODIS), available since 2000, have also shown accelerated snow losses at extratropical latitudes and mid-latitudes. According to MODIS estimates over the period 2000–2016, the annual SC extent shrunk about 13% around latitude 34°S^22^ while the annual snow persistence declined at a rate of 10–20% per decade at latitudes 23°–40°S^23^.

Decreasing snow trends may be partially attributed to increases in surface air temperature^24^. However, analyses of global trends have shown that, at high elevations, precipitation has greater relative importance on snow persistence^8^. In the case of the Andes, despite the relevant influence of the temperature (especially at tropical latitudes and at mid-latitude lower elevations), precipitation has been found to be the main driver of MODIS-based snow losses^23^.

The interannual variability of precipitations in our study area (18°–40°S) is determined by large-scale modes such as the El Niño-Southern Oscillation (ENSO) and the Southern Annular Mode (SAM). The ENSO warm phase (El Niño) is associated with positive sea surface temperature (SST) anomalies in the tropical Pacific Ocean, which enhances precipitations^25,26^ and snow accumulation^27^ in the Andean region. ENSO is well correlated with the Andean snow cover at tropical/extratropical latitudes while the SAM influence is more important at higher latitudes^8,23^. SAM is characterized by a latitudinal vacillation of the tropospheric-deep westerly wind maxima around 50°S, which modulates the precipitation regime over the southeast Pacific^28^.

In recent decades the ENSO amplitude has weakened on average and their SST anomalies shifted westward towards the central Pacific^29,30^. The causes of these changes remain under debate, but they are likely related to the changing background conditions in the tropical Pacific Ocean^31^. Moreover, both greenhouse gas emissions and the ozone depletion have induced a robust trend toward the positive phase of SAM^32–34^. The poleward migration of the westerly winds (associated with the positive phase of SAM) has led to precipitation drops at Andean mid-latitudes^35^, which has likely affected the SC extent in the area.

Although the influence of El Niño and SAM on the interannual SC *variability* is well established^8,21–23^, the role of these large-scale modes (subjected to significant changes in recent decades) in the Andean SC *losses* has not been stated.

**Figure 1:**
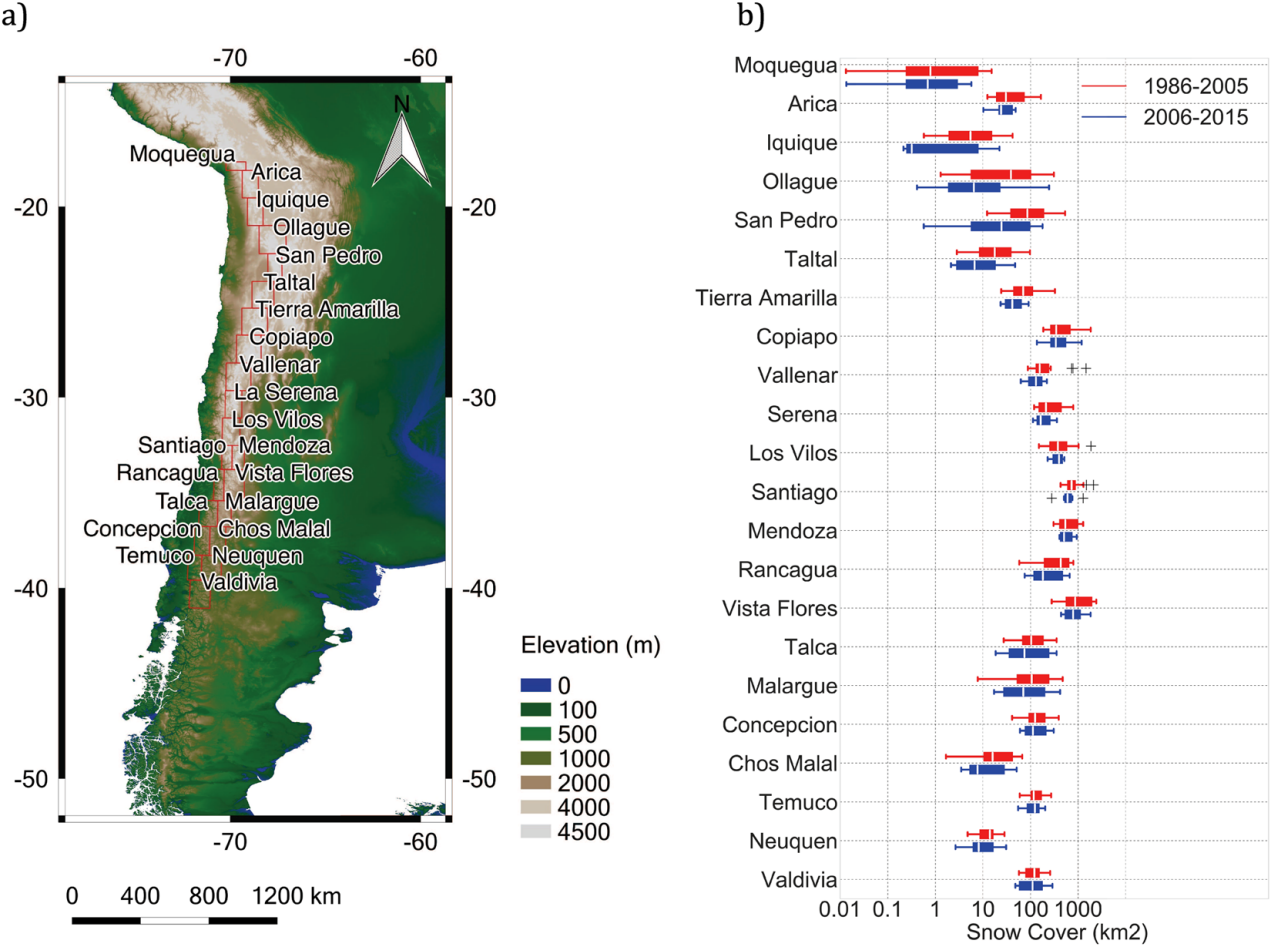
(**a**) Map of the study area. The dry-season snow cover extent was retrieved from Landsat imagery over the 22 zones indicated by the rectangles. Names used to identify these zones were quoted from cities or towns nearby. Topographic information for this plot was obtained from the CIAT-CSI SRTM website^67^. (**b**) Boxplot of the dry-season snow cover extent over the period 1986–2005 (red) and over the period 2006–2015 (blue), in the 22 zones indicated in (**a**). In each box, the central mark (white stripe) indicates the median, and the edges indicate the 25th and 75th percentiles. The whiskers extend to the most extreme data points not considered outliers, and the outliers are plotted individually using the ‘+’ symbol. Plots were generated by using PYTHON’s Matplotlib Library^68^.

Here we use Landsat imagery over the period 1986–2018 in order to assess changes in the dry-season SC extent across a north-south transect of approximately 2,500 km in the Andes (18°–40°S). Due to the different climate regimes, we have split our study area into 3 macrozones: tropical latitudes (18°–23°S), extratropical latitudes (23°–34°S), and mid-latitudes (34°–40°S). Since the approximate Landsat scene size is 170 km north-south by 183 km east-west, these 3 macrozones were further subdivided into the 22 rectangular zones shown in Fig. 1a. Names used to identify these zones were adopted from cities or towns nearby. Geographic coordinates of the 22 zones are shown in Table S1.

We focused on the dry-season within which, according to prior efforts^35,36^, precipitation in our study area has shown significant anomalies in recent decades. From latitude 18°S to latitude 23°S (in the northern Atacama Desert), the dry-season occurs from September to December (in what follows SOND)^37^; from latitude 23°S to latitude 40°S, the dry-season roughly occurs during the austral summer (January, February, March; JFM)^38,39^.

## Results

### SC losses

Figure 1b shows the boxplot of the dry-season SC extent retrieved from Landsat imagery over the 22 zones indicated in Fig. 1a, over two periods: 1986–2005 (red) and 2006–2015 (blue). As shown in Fig. 1b, the dry-season SC extent in the northern Atacama Desert (18°–23°S) tends to be significantly lower than in extratropical zones (23–34°S). The relatively low SC values from latitude 18°S to latitude 23°S are consistent with the fact that in the Tropics, Andean snow is constrained to high elevations (>5000 m)^8,9^. Due to higher precipitation and lower temperatures (compared to the northern Atacama Desert)^39^, the Andean dry-season SC peaks at extratropical latitudes (23°–34°S), then slightly decreasing again at mid-latitudes (34°–40°S).

Figure 1b also allows assessing the significant losses in the SC extent in recent decades across the entire study area. Comparing the boxes computed over the periods 1986–2005 (red) and 2006–2015 (blue), it can be observed that the distribution of the SC retrievals shifted toward lower values in almost all zones. This shifting has increased the likelihood of low SC values during the dry-season. According to Fig. 1b, in each of the 22 zones within our study area (especially in the northern Atacama Desert), low dry-season SC values are significantly more likely nowadays than over the base period 1986–2005.

Figure 1b shows that the dry-season SC extent declined in recent decades in our study area: about 39% at tropical latitudes (18°–23°S), and more than 19% at extratropical and mid-latitudes (23°–40°S). Note that these changes are likely influenced by the drought that affected the region since 2010^40^.

### Correlation with climate indices

**Figure 2:**
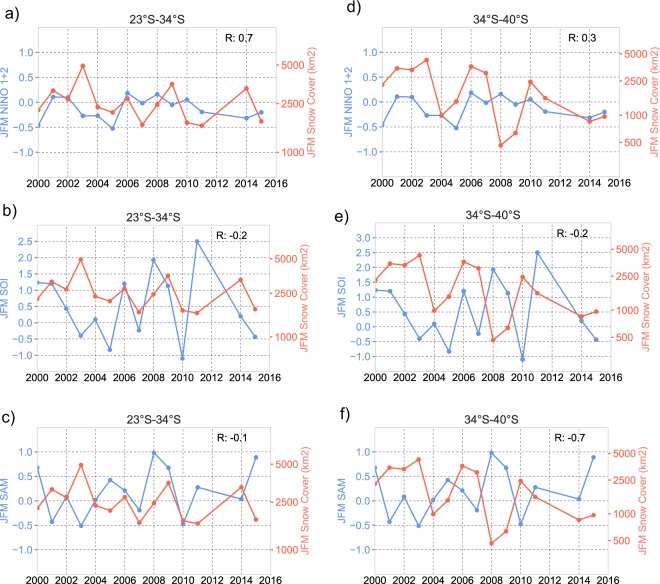
Time series of the dry-season SC extent and the time series of the SST anomaly in the Niño 1 + 2 region (first row), the Southern Oscillation Index (SOI) values (second row), and the Southern Annular Mode (SAM) index (third row). (**a**–**c**) Extratropical latitudes (23°S–34°); and (**d**–**f**) Mid-latitudes (34°S–40°). The correlation coefficients (R) are shown in the upper right corner of each plot. The plots were generated by using PYTHON’s Matplotlib Library^68^.

We tested the correlations between the dry-season SC extent (in the 3 macrozones considered in this study) and 3 climate indices: the SST anomaly in the Niño 1 + 2 region (0–10°S, 90°W–80°W)^41^, the Southern Oscillation Index (SOI)^42^, and the SAM index^43^. Weekly SST anomalies in the Niño regions were provided by NOAA’s Climate Prediction Center (CPC): https://www.cpc.ncep.noaa.gov/data/indices/wksst8110.for; monthly SOI values were provided by NOAA’s National Center for Environmental Information: https://www.ncdc.noaa.gov/teleconnections/enso/indicators/soi/data.csv; and monthly SAM data were provided by NOAA’s Earth System Research Laboratory: https://www.esrl.noaa.gov/psd/data/20thC_Rean/timeseries/monthly/SAM/. Figure 2 shows the time series of the dry-season SC extent (averaged over each of the 3 macrozones considered in this study) and the time series of the SST anomaly in the Niño 1 + 2 region (first row), the SOI values (second row), and the SAM index (third row). The correlation coefficients (R) are shown in the upper right corner of each plot.

Figure 2 confirms a relatively good correlation between the dry-season SC extent at extratropical latitudes and the SST anomaly in the Niño 1 + 2 region (see Fig. 2a). This is not surprising since the Niño 1 + 2 region corresponds to the equatorial Pacific coast of South America^41^. The correlation between ENSO/El Niño indices and the Andean SC extent at tropical/extratropical latitudes has been widely confirmed^8,20,22^. The strong correlation between the Andean snow persistence and SST anomalies in the Niño 1 + 2 region has been also previously highlighted^23^. As shown in Fig. 2d, the correlation between the dry-season SC extent and the SST anomaly in the Niño 1 + 2 region is lower at higher latitudes (34°–40°S).

The correlation between the SOI values and the dry-season SC extent is not particularly strong (see Fig. 2b,e). The SOI value depends on the observed sea level pressure differences between Darwin (Australia) and Tahiti^42^. The SOI corresponds to the fluctuations in air pressure between the eastern and western tropical Pacific during the ENSO phases^42^. A stronger correlation between the SOI values and the snow persistence at extra-tropical latitudes has been previously reported^23^ but that involved a different time scale.

Figure 2 also confirms a relatively high correlation between the SAM index and the dry-season SC extent at mid-latitudes (see Fig. 2f). The SAM index is defined as the difference of zonal mean sea level pressure between 40°S and 65°S^43^; a positive index (lower polar pressure) is associated with weaker zonal winds while a negative value is associated with stronger zonal winds^43^. The SAM influence on the SC extent at latitudes higher than 34°S was expected since the westerlies modulate the precipitation regime over the southeast Pacific^28^. The good correlation between the SC at mid-latitudes and the SAM index has also been reported in prior efforts^8,23^.

### SC trends

**Figure 3:**
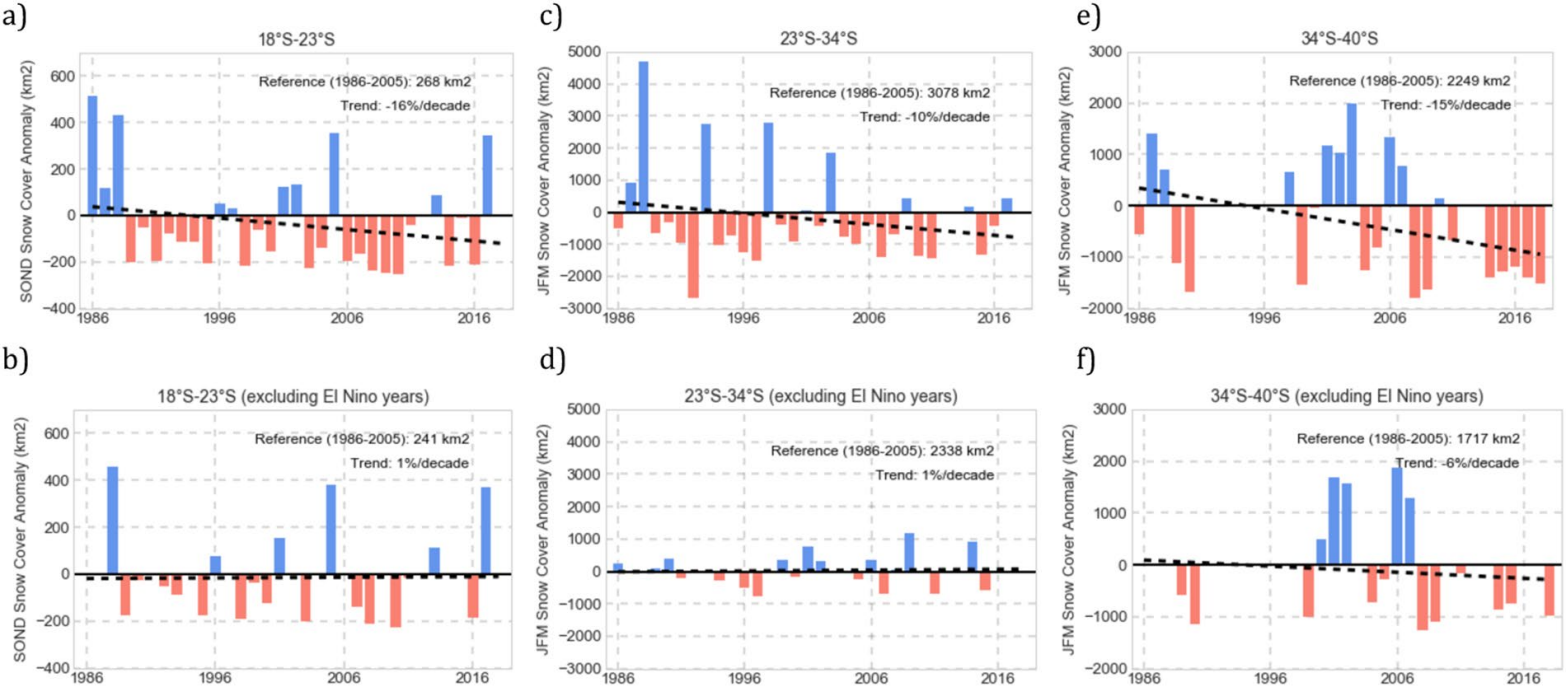
First row: Dry-season snow cover anomalies over the period 1986–2017/8. Second row: the same as in the first row but excluding El Niño years. (**a**,**b**) Tropical latitudes (18°S–23°); (**c**,**d**) Extratropical latitudes (23°S–34°); and (**e**,**f**) Mid-latitudes (34°S–40°). The linear regression trendline is shown in each plot. The trend is shown in the upper right corner of each plot as well as the mean of the dry-season snow cover extent computed over the base period 1986–2005 (this was the reference value used for computing the anomalies and trends). Our findings are based on a total of 1952 Landsat images acquired under cloudless conditions over the period 1986–2017 from latitude 18°S to latitude 33°S, and over the period 1986–2018 from latitude 33°S to latitude 40°S. Note that due to the fewer Landsat scenes available for some zones at mid-latitudes (34°–40°S), anomalies over the period 1991–1996 were neither included in plots (**e**,**f**) nor used for computing the trends in this area. The plots were generated by using PYTHON’s Matplotlib Library^68^.

Figure 3 (first row) shows the dry-season SC anomalies computed for our 3 macrozones: tropical latitudes (18°–23°S), extratropical latitudes (23°–34°S), and mid-latitudes (34°–40°S). As shown in Fig. 3 (first row), the dry-season SC extent exhibits decreasing trends in each of our 3 macrozones: about −16% per decade at tropical latitudes (18°–23°S), approximately −10% per decade at extratropical latitudes (23°–34°S), and about −15% per decade at mid-latitudes (34°–40°S). The significance of these trends was tested by using the Mann-Kendall (M-K) test^44^. Despite the interannual variability, the trends are statistically significant (see Table 1), especially at tropical latitudes (18°–23°S) and at mid-latitudes (34°–40°S).

**Table 1:** Dry-season snow cover trend **(**and the corresponding M-K test significance) computed from Landsat scenes acquired under cloudless conditions over the period 1986–2017 from latitude 18°S to latitude 33°S, and over the period 1986–2018 from latitude 33°S to latitude 40°S.

	Trend (%/decade) over the period 1986–2017/8	Significance of the trend (according to the M-K Test
Tropical Latitudes (18°–23°S)	−16	at 0.1 level
Extra-Tropical Latitudes (23°–34°S)	−10	at 0.4 level
Mid-Latitudes (34°–40°S)	−15	at 0.1 level

Snow trends in the macrozones considered in this study are appreciably different when El Niño years are not considered. As shown in Fig. 3 (second row), excluding “El Niño years” (see the section “Methods” for details on the adopted definition of “El Niño years”), dry-season SC extent exhibits no significant trend at tropical/extratropical latitudes (see plots 3b and 3d), while the decreasing trend at mid-latitudes is cut roughly in half (see plot 3 f). We did not find significant effects associated with La Niña in the SC extent in our study area. However, this is consistent with prior efforts that have shown a weak relation between La Niña and the snow accumulation over the Andes^27^.

The interannual variability also is appreciably different with/without considering El Niño years. This can be noted when comparing Fig. 3c,d (plotted deliberately using the same plot range); anomalies in the dry-season SC extent are significantly lower when excluding El Niño years (Fig. 3d). The interannual variability (taken as the standard deviation of the annual dry-season SC averages over the period 1986–2018) dropped at extratropical latitudes (23°–34°S) by about 50% when excluding El Niño years with respect to the *all year-included* variability. The variability drop is less significant in the case of tropical latitudes (18°–23°S) and mid-latitudes (34°–40°S).

**Figure 4:**
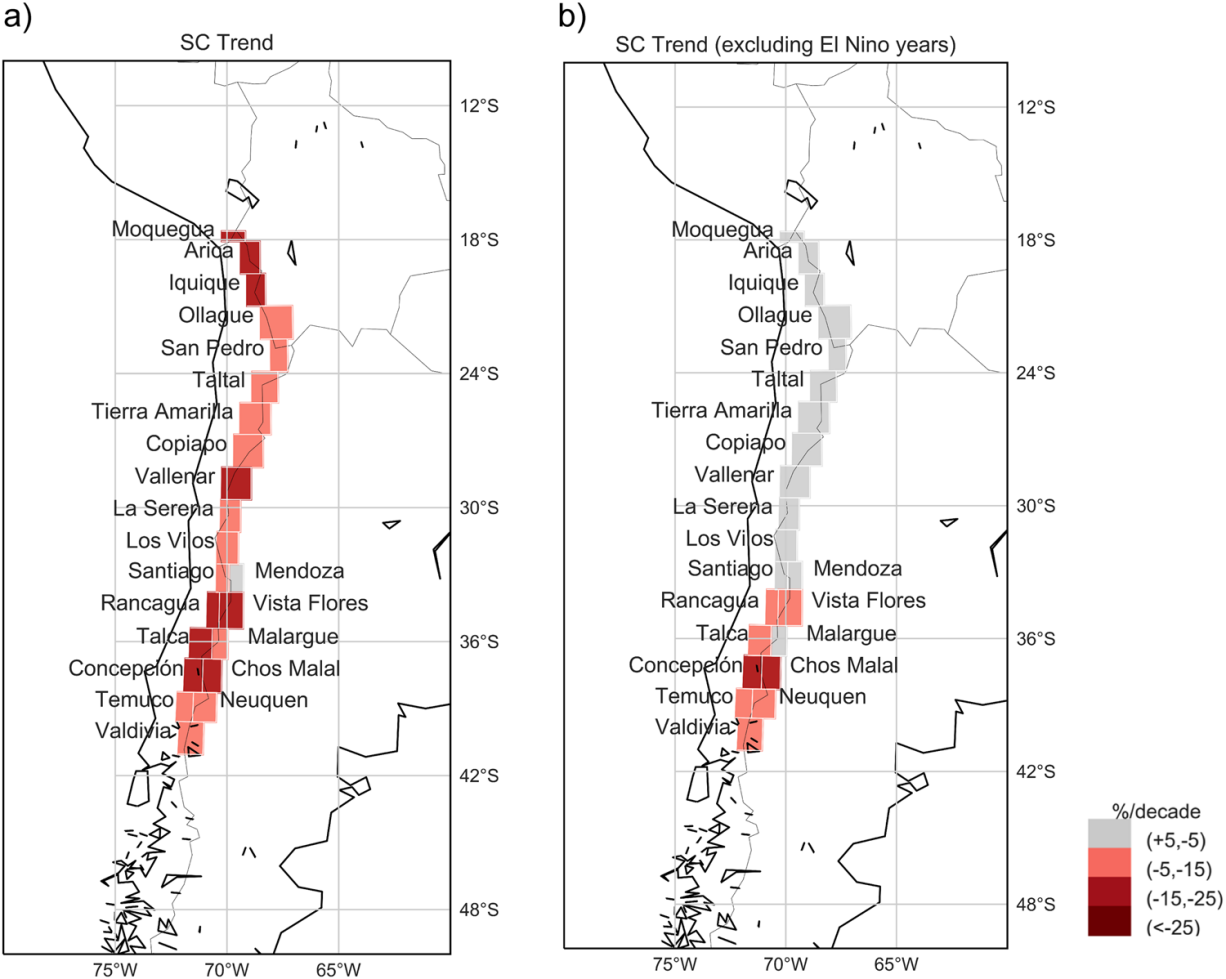
(**a**) Dry-season snow cover trend computed over the period 1986–2017/8. (**b**) The same as in (**a**) but excluding El Niño years. Changes are relative to the mean dry-season snow cover over the base period 1986–2005. The plots were generated by using PYTHON’s Matplotlib Library^68^.

Trends for each of the 22 zones within our study area are shown in Fig. 4. Figure 4a shows the *all years-included* dry-season SC trend computed over the period 1986–2018. Figure 4a allows highlighting some of the differences between west and east sides of the Andes, especially at mid-latitudes (34°–40°S). Differences were expected since prior efforts have reported contrasting precipitation regimes on both sides of the Andes^45^. As shown in Fig. 4a, snow losses tend to be greater on the western side than on the eastern side of the Andes. However, these differences were significant only between “Santiago” and “Mendoza” as well as between “Talca” and “Malague”; names used to identify these 4 zones were quoted from cities or towns nearby. The differences at mid-latitudes are likely influenced by the dissimilar effects of the westerly winds on the precipitations on each side of the Andes^28^.

Figure 4b shows the dry-season SC over the same period but without considering El Niño years. When comparing Fig. 4a,b, it can be observed that snow trends are appreciably different with/without considering El Niño years. Without considering El Niño years (see Fig. 4b), the SC trends drastically shrunk in all of the 22 zones within our study area exhibiting no significant trends at tropical/extratropical latitudes (18°–34°S). However, a *non-El Niño years* SC trend is still observed at mid-latitudes (34°–40°S) in Fig. 4b, which is likely related to the drying of this area observed in recent decades^35,36^.

At Andean mid-latitudes, a decreasing precipitation trend has been observed in data collected from 1960 to 2016 by rain gauges along the Pacific coast of South America^36^. Part of this trend has been attributed to the weakening of the SH westerly winds around 40°S^35^, which is in turn linked with a robust trend toward the positive phase of the SAM^32–34^.

**Figure 5:**
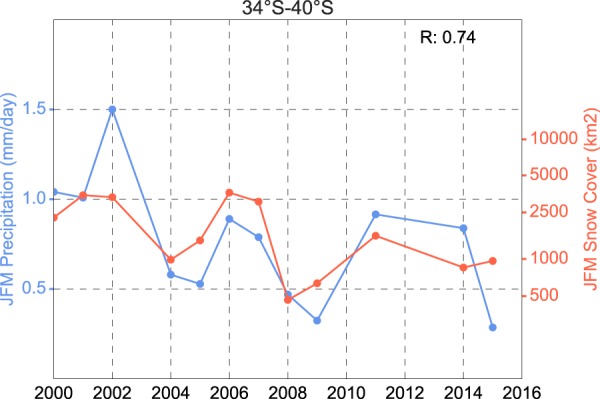
Time series of annual mean of the dry-season (JFM) snow cover extent (red line) and annual mean of the dry-season (JFM) daily precipitation (blue line). These data were averaged over the Andean mid-latitudes (34°S–40°). El Niño years were excluded and snow cover data were not available for 2012 and 2013. The correlation coefficient (R) is shown in the upper right corner. Precipitation data were obtained from the Global Precipitation Climatology Project (GPCP) Version 2.2 Combined Data Set^69^. The plot was generated by using PYTHON’s Matplotlib Library^68^.

Figure 5 shows a strong correlation (R = 0.74) between the annual mean of the dry season SC extent (red line) and the annual mean of the dry-season daily precipitations (blue line). Data were averaged over the Andean mid-latitudes (34°S–40°) and El Niño years were excluded. Although the correlation between the SAM index and the snow persistence at Andean mid-latitudes was already known^8,9^, both Figs 2f and 5 highlight the role of SAM-related precipitation changes in the SC losses at the Andean mid-latitudes (34°–40°S). Note that the insignificant influence of the SAM trend on the SC losses at lower latitudes is consistent with prior efforts that found that the role of SAM on the non-ENSO precipitation regime at Andean extratropical latitudes was secondary^46^.

### Dataset consistency

In this study, we analyzed a total of 1952 Landsat images acquired under cloudless conditions over the period 1986–2017 from latitude 18°S to latitude 33°S, and over the period 1986–2018 from latitude 33°S to latitude 40°S. Dry-season snow cover averages for each macrozone are based on hundreds of Landsat scenes. More than 400 (200) images were analyzed over the period 1986–2005 (2006–2015) in each of the 3 macrozones: tropical latitudes (18°–23°S), extratropical latitudes (23°–34°S), and mid-latitudes (34°–40°S).

Dry-season SC trends for each of the 3 macrozones within our study area (see Fig. 3) were computed using more than 500 Landsat scenes per macrozone. However, dry-season SC trends for each of the 22 zones within our study area (see Fig. 4) were computed using fewer images; typically less than 100 Landsat scenes were available per zone. Nevertheless, trends obtained when analyzing the SC anomalies separately in these 22 zones were found to be consistent with the trends in the 3 macrozones within our study area.

Figure S1 (first row) shows the dry-season SC anomalies computed for the zones that accounted for most of the typical snow cover in each of the 3 macrozones. Figure S1 (second row) shows the corresponding dry-season SC anomalies without considering El Niño years.

At tropical latitudes, the *all years-included* dry-season SC trend ranged in Fig. 4a from −8%/decade in “Ollague” to −21%/decade in “Iquique”. However, the SC extent in the northern Atacama Desert (18°–23°S) is driven by “San Pedro”, which accounts for more than half of the typical average area covered by snow in this area. This explains the good agreement between the SC trends computed (with/without considering El Niño years) for the Andean tropical latitudes (see Fig. 3a,b) and for “San Pedro” (see Fig. S1a,b).

At extratropical latitudes, the *all years-included* dry-season SC trend in Fig. 4a ranged from −5%/decade in “Mendoza” to −21%/decade in “Vallenar”. Yet, the SC extent at extratropical latitudes is driven by “Santiago” and “Copiapo”, which together account for about half of the typical average area covered by snow in this area. This explains why the SC trends computed (with/without considering El Niño years) for the Andean extratropical latitudes (see Fig. 3c,d), roughly agree with SC trends computed for “Copiapo” (see Fig. S1c,d) and for “Santiago” (see Fig. S1e,f).

At mid-latitudes, the *all years-included* SC trend in Fig. 4a ranged from −11%/decade in “Neuquen” to −21%/decade in “Chos Malal”. However, fewer Landsat scenes were available over Andean mid-latitudes, especially for the 1990s. In general, years with less than 3 Landsat scenes available per season, per zone, were not used for computing anomalies/trends. Since this was the case for several zones at Andean mid-latitudes (34°–40°S) during the 1990s, anomalies over the period 1991–1996 were not included in Fig. 3e,f. Still, we did have a reasonable number of Landsat scenes available in the case of “Vista Flores”, which accounts for more than half of the typical average area covered by snow at Andean mid-latitudes (34°–40°S). We found a good consistency between the SC trends computed (with/without considering El Niño years) for “Vista Flores” (see Fig. S1g,h) and for the Andean mid-latitudes (see Fig. 3e,f).

## Discussion

Our results show that the dry-season SC extent has declined over the period 1986–2018 across the entire study area. Although the detected SC changes are likely beyond the expected natural variability, they may be partially influenced by the Pacific Decadal Oscillation (PDO). The PDO is a long-lived El Niño-like pattern, whose phase changes over a longer period^47–49^. Although the interdecadal precipitation variability in central Chile (30-43°S**)** is correlated with the PDO^50^, no distinguishable PDO-related signatures have been found in the Chilean and Argentinean snowpack series over the period 1941–2008^51^.

Decreasing snow trends were also found across the entire study area. The dry-season SC extent declined at a rate of about −16% per decade at tropical latitudes (18°–23°S), approximately −10% per decade at extratropical latitudes (23°–34°S), and about −15% per decade at mid-latitudes (34°–40°S). These decreasing trends hold up despite the interannual variability, but they are appreciably different with/without considering El Niño years. Without considering El Niño years, dry-season SC extent exhibits no significant trend at tropical/extratropical latitudes (18°–34°S) while the decreasing trend at mid-latitudes (34°–40°S) is cut roughly in half.

Our results suggest that changes in El Niño likely accounts for most of the Andean SC *losses* at tropical/extratropical latitudes (18°–34°S). El Niño determines the SST anomalies of the equatorial Pacific Ocean, which in turn strongly affects precipitation in the Andean region. Over the past two decades, El Niño events have weakened on average and their SST anomalies shifted westward towards the central Pacific^29,30^. Due to the correlation between the eastern equatorial Pacific (the Niño 1 + 2 region) SST anomalies and the Andean snow persistence^23^, the westward shifting of the equatorial SST anomalies likely contributed to the SC losses in the Andean region in recent decades. It is uncertain if El Niño-related snow losses will continue at similar rates in the future in the Andean region (especially at tropical/extratropical latitudes), because it is unknown how the ENSO may change under future greenhouse warming^52^. Climate models show that under a likely emission scenario, extreme El Niño frequency increases linearly with the global mean temperature towards a doubling at 1.5 °C warming^53^. These increases in the number of ‘strong’ El Niño events are associated with large SST anomalies in the eastern equatorial Pacific (Niño 1 + 2 region)^52^. Although it is uncertain how these extreme El Niño events may affect the Andean SC trend, they will likely boost the interannual SC variability.

In addition to El Niño, our results suggest that dry-season snow losses at Andean mid-latitudes (34°–40°S) are also influenced by the trend toward the positive phase of the SAM during spring and summer observed in recent decades^32–34^. The SAM trend is associated with a poleward migration of the westerly winds that appears to be driven by both greenhouse gas emissions and the ozone depletion^34^. The latter affects the frequency of extreme anomalies and the persistence of SAM^54^. Although the effect of the ozone depletion is expected to reduce as stratospheric ozone recovers to pre ozone hole levels, a robust positive trend in the SAM is projected under high greenhouse gas emission scenarios^55^. This may result in further SC losses at Andean mid-latitudes in the future.

## Methods

### SC data

We used data from three sensors: Landsat Thematic Mapper (TM) carried on Landsat 5, Landsat Enhanced Thematic Mapper Plus (ETM+) carried on Landsat 7, and Landsat 8 Operational Land Imager (OLI). Imagery is available since 1986, 2000, and 2013, for Landsat 5 TM, Landsat 7 ETM+, and Landsat 8 OLI, respectively. In each case, images consist of several spectral bands with a spatial resolution of 30 meters for both the green band (0.52–0.60 μm) and the short-infrared band (1.55–1.75 μm), which were used for computing the Normalized Difference Snow Index (NDSI) values^56^. The approximate scene size is 170 km north-south by 183 km east-west. Landsat satellites have relatively short cycles (16 days)^57^. The images were provided by the United States Geological Survey (USGS): https://earthexplorer.usgs.gov/.

All Landsat scenes were projected to the World Geodetic System (WGS84), zone 19 south. The scenes were projected using a raster for geographic data analysis and modeling embedded in R’s software package^58,59^. Next, we converted Landsat digital numbers (DN) to the top-of-atmosphere (TOA) radiance and applied a dark object subtraction (DOS)-based atmospheric correction to convert TOA radiance to surface reflectance^60^. For this procedure, we used the tools radCor and sdos included in the RStoolbox, a set of tools for remote sensing data analysis^61^.

The DOS-based atmospheric correction allowed us to work with data expressed in actual physical units (i.e. reflectance). Atmospheric correction is particularly important for mapping methods that utilize bands in the visible spectrum (such as NDSI)^62^. This correction enabled us to standardize scenes from different dates and/or sensors.

After atmospherically correcting each scene, we also applied the Minnaert correction to account for topographic effects on illumination^61^ and computed the SC surface by using the SAGA tool included in the QGIS Geographic Information System^63^. The Shuttle Radar Topography Mission (SRTM) 30 m digital elevation model (DEM) provided by USGS (https://earthexplorer.usgs.gov/) was used.

Following prior efforts^64^, the SC extent was mapped according to a binary identification based on a single threshold for the NDSI data^56^. NDSI-based methods have been found to be effective in deeply shadowed areas such as those often found over the Andes^64^. The NDSI takes the difference between the green band (0.52–0.60 μm) and the short-infrared band (1.55–1.75 μm) divided by the sum of those two bands^65^. NDSI values tend to be higher over fresh snow than over wet old/shadowed snow.

According to prior efforts^66^, the threshold value in our case was selected after making a visual comparison between SC mapped with a certain threshold and a color- or false-color- composite image from the same scene. The threshold value (0.4) was applied to all NDSI images, which were derived from atmospherically-corrected Landsat scenes acquired under cloudless conditions. Obvious mapping errors, such as lakes, were masked.

We tested the effect of the different topographic corrections and slightly different NDSI thresholds (0.35 to 0.55) on our results. We compared SC estimates generated using uncorrected scenes and corrected scenes as well as SC estimates generated using slightly different NDSI thresholds. Our tests suggest that, although the selection of thresholds and topographic corrections has an impact on snow area estimates, neither has significant effects on the SC trends.

### El niño years

NOAA’s Climate Prediction Center (CPC) considers that an El Niño episode is characterized by a five consecutive 3-month running mean of SST anomalies in the Niño 3.4 region (5°N-5°S, 170°W–120°W), that is above the threshold of +0.5 °C^41^. However, as shown in Fig. 2 (first row), the SST anomaly in the Niño 1 + 2 region (0–10°S, 90°W–80°W) is also very important in the Andean region.

In order to compute *non-El Niño years* SC trends, we excluded the SC anomalies corresponding to the years 1986, 1987, 1991, 1994, 1997, 2002, 2004, 2006, 2009, 2014, and 2015 in Fig. 3b. All these years were considered El Niño years by NOAA’s CPC. However, more importantly, we confirmed that the weekly SST anomalies in the Niño region 1 + 2 were above the threshold of +0.5 °C during SOND in those years (the dry-season in the northern Atacama Desert). In the case of Fig. 3d,f, we excluded the SC anomalies corresponding to the years 1987, 1988, 1992, 1993, 1995, 1998, 2003, 2010, 2016, and 2017. Except for 1993 and 2017, all these years were considered El Niño years by NOAA’s CPC. The years 1993 and 2017 were excluded because the JFM average of the weekly SST anomalies in the Niño 1 + 2 region was above the +0.5 °C threshold in those years. Weekly SST anomalies in the Niño regions were provided by NOAA’s CPC: https://www.cpc.ncep.noaa.gov/data/indices/wksst8110.for.

## Supplementary information

Supplementary 1

## Data Availability

The Shuttle Radar Topography Mission (SRTM) 30 m digital elevation model (DEM) as well as the images from Landsat Thematic Mapper (TM) carried on Landsat 5, Landsat Enhanced Thematic Mapper Plus (ETM+) carried on Landsat 7, and Landsat 8 Operational Land Imager (OLI), were provided by the United States Geological Survey (USGS): https://earthexplorer.usgs.gov/. Weekly sea surface temperature (SST) anomalies in the Niño regions were provided by NOAA’s Climate Prediction Center (CPC): https://www.cpc.ncep.noaa.gov/data/indices/wksst8110.for; monthly southern oscillation index (SOI) values were provided by NOAA’s National Center for Environmental Information: https://www.ncdc.noaa.gov/teleconnections/enso/indicators/soi/data.csv; and monthly southern annular mode (SAM) data were provided by NOAA’s Earth System Research Laboratory: https://www.esrl.noaa.gov/psd/data/20thC_Rean/timeseries/monthly/SAM/. We used precipitation data “GPCP Version 2.2 Combined Data Set”, available at https://precip.gsfc.nasa.gov/gpcp_v2.2_comb_new.html. Additional datasets are available from the corresponding author on reasonable request.
