# Supplementary material for: Dry-Season Snow Cover Losses in the Andes (18°–40°S) driven by Changes in Large-Scale Climate Modes
Source: Sci Rep. 2019 Nov 18;9:16945. doi: 10.1038/s41598-019-53486-7 (PMC6861277; doi:10.1038/s41598-019-53486-7)
Supplement: Supplementary file 1 — Supplementary 1 [file 41598_2019_53486_MOESM1_ESM.pdf]

Supplementary material

Dry-Season Snow Cover Losses in the Andes (18°-40°S) driven by Changes in Large-Scale Climate Modes

Raul R. Cordero[1], Valentina Asencio[1], Sarah Feron[2,*], Alessandro Damiani[3], Pedro J. Llanillo[1], Edgardo Sepulveda[1], Jose Jorquera[1], Jorge Carrasco[4], Gino Casassa[4,5]

1. Universidad de Santiago de Chile, Av. Bernardo O'Higgins 3363, Santiago, Chile
2. School of Earth, Energy and Environmental Sciences, Stanford University, Stanford, California, USA
3. Center for Environmental Remote Sensing, Chiba University, Chiba, Japan
4. Centro de Investigación GAIA Antártica, Universidad de Magallanes, Punta Arenas, Chile
5. Unidad de Glaciología y Nieves, Dirección General de Aguas, Ministerio de Obras Públicas, Santiago, Chile

* Correspondence to sferon@stanford.edu

Table S1. Zones where the snow cover extent was assessed. Names used to identify these zones were adopted from cities or towns nearby.

		West	East	North	South
Tropical Latitudes (18°S-23°S)	Moquegua	70.22°W	69.17°W	18.08°S	17.62°S
	Arica	69.37°W	68.52°W	19.52°S	18.08°S
	Iquique	69.09°W	68.27°W	20.97°S	19.52°S
	Ollague	68.49°W	67.05°W	22.44°S	20.97°S
	SanPedro	68.02°W	67.28°W	23.89°S	22.44°S
Extra-Tropical Latitudes (23°S-34°S)	Taltal	68.86°W	67.7°W	25.30°S	23.89°S
	TierraAmarilla	69.39°W	68.03°W	26.72°S	25.30°S
	Copiapo	69.67°W	68.37°W	28.19°S	26.72°S
	Vallenar	70.21°W	68.91°W	29.63°S	28.19°S
	Serena	70.26°W	69.39°W	31.08°S	29.63°S
	Vilos	70.41°W	69.50°W	32.52°S	31.08°S
	Santiago	70.43°W	69.91°W	33.78°S	32.52°S
	Mendoza	69.90°W	69.27°W	33.78°S	32.52°S
Mid-Latitudes (34°S-41°S)	Rancagua	70.84°W	70.35°W	35.40°S	33.77°S
	VistaFlores	70.65°W	69.96°W	35.40°S	33.77°S
	Talca	71.62°W	70.68°W	36.76°S	35.38°S
	Malargue	70.65°W	69.96°W	36.76°S	35.38°S
	Concepción	71.85°W	71.10°W	38.27°S	36.74°S
	Chos Malal	71.06°W	70.25°W	38.27°S	36.74°S
	Temuco	72.19°W	71.53°W	39.58°S	38.24°S
	Neuquen	71.49°W	70.49°W	38.58°S	38.24°S
	Valdivia	72.10°W	71.07°W	41.04°S	39.56°S

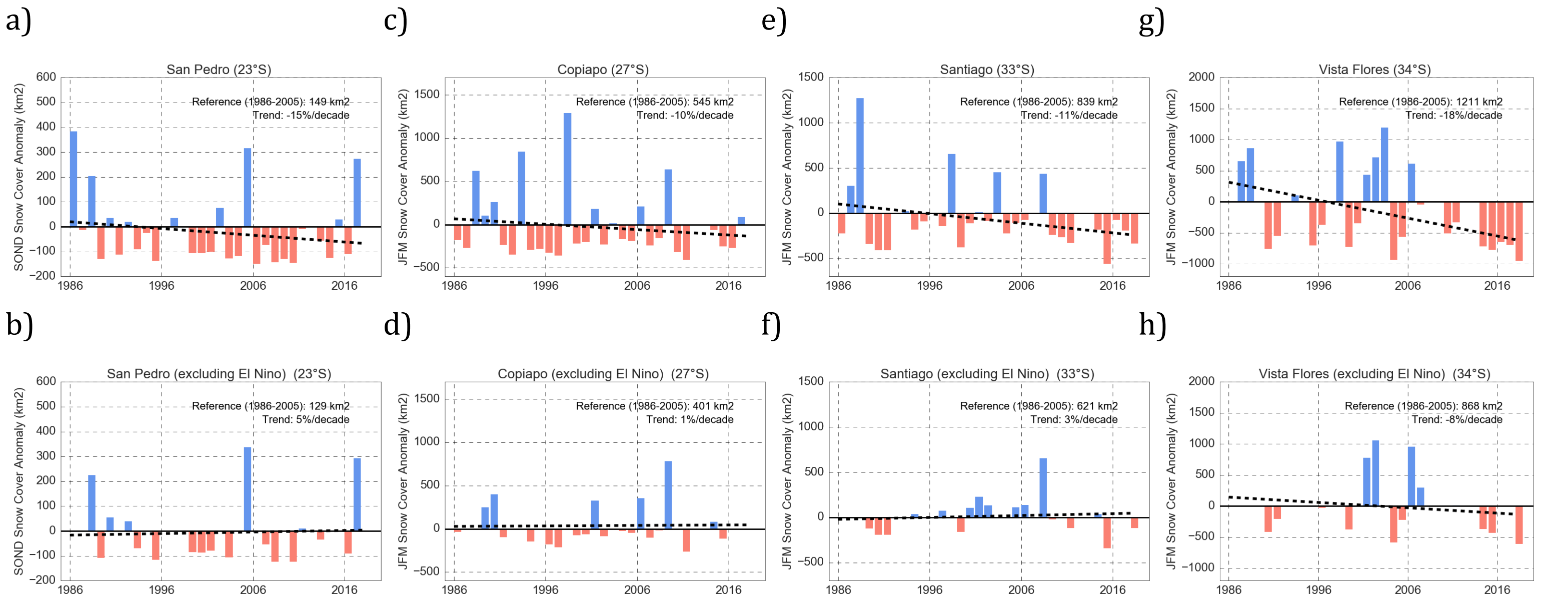

Figure S1. First row: dry-season snow cover anomalies computed for the zones that accounted for most of the typical snow cover in our study area. Second row: the same as in the first row but excluding El Niño years. a,b) San Pedro; c,d) Copiapo; e,f) Santiago; g,h) Vista Flores. The linear regression trendline is shown in each plot. In the upper right corner of each plot the trend as well as the mean dry-season snow cover over the period 1986–2005 (corresponding to the reference value used for computing anomalies and trends) are shown. The plots were generated by using PYTHON's Matplotlib Library[68].
